# Epithelioma Treated with Arsenic

**Published:** 1885-12-20

**Authors:** J. B. Medlock

**Affiliations:** Georgia


### EPITELIOMA TREATED WITH ARSENIC.

By J. B. MEDLOCK, M. D., OF GEORGIA.

I will report a case in which those, at least, who have been writing on that subject may find interest, viz: the use of arsenic in malignant disease.

About the first of August was called to see Mrs. G——, age fifty, on account of a small tumor situated on inferior labia, opposite the right canine tooth. Patient is a tall lady, and presents the cancerous cachexia; is the more alarmed because her mother died of a tumor on the nose. The tumor looked red, and a little constricted at base; presents, on all sides, a cauliflower protuberance, from which exudes a bloody, fetid serum; has been cauterized repeatedly by a regular physician, who, finding no relief from local applications, proposed the knife, which proposition caused the poor sufferer to seek "anothor doctor."

Having Dr. Cotton in consultation with me in another case at the time, I asked him to examine the case for me, and we at once decided that it was a case of epithelioma. I decided at that time to try the arsenical treatment, and prescribed, as a local dressing, carbolic acid and vaseline, and ordered the bowels moved with pulv. rheubarb This was done each day until the digestive organs were in tolerably good condition. I then prescribed arsenious acid, one-eighth grain in coffee, before breakfast, and ordered a dose of rheubarb at 10 o'clock, if needed to move the bowels.

This treatment—to be brief—was continued four weeks, the dose being gradually increased so that, at the end of the first month, one-half grain was taken daily. The tumor had now ceased to grow, and I removed it with a catgut ligature. I continued the local treatment one week, when only a very small cicatrix was left to show the site of the tumor. Now I began to decrease the dose of arsenic gradually, and every third or fourth dose was omitted, so that at the end of the second month of treatment the dose was one-eighth of a grain. I am now, at the end of the third month, giving small doses of the medicine, and occasionally as the constitutional symptoms—viz, increased secretion from bowels and kidneys—begin to appear, omit it.

I shall soon suspend the remedy for a while, as the appetite is good, and the health of my patient is better than for months before the beginning of the above treatment.

I neglected, in giving the very brief history of the case, to state that the tumor had been growing about two months when I was first consulted.

I hope that the history and treatment of this case may not lead to harm, by causing any one to be too careless in the administration of this powerful drug; so I would advise any one, especially the young and inexperienced, as myself—having been in the practice but two years—to see his patient at least once, better twice, every day, and watch the bowels and the effects of this medicine. With proper care, I believe this to be a safe and efficient mode of treatment in this class of diseases.

